# Antioxidant-Enzyme Profiles in Youth Athletes: Associations of SOD and GPX with Exercise and Implications for Endothelial Health

**DOI:** 10.3390/ijms26199532

**Published:** 2025-09-29

**Authors:** Jonas Haferanke, Sebastian Freilinger, Lisa Baumgartner, Tobias Engl, Maximilian Dettenhofer, Stefanie Huber, Frauke Mühlbauer, Renate Oberhoffer, Thorsten Schulz

**Affiliations:** Institute of Preventive Pediatrics, Department Health and Sport Sciences, TUM School of Medicine and Health, Technical University of Munich (TUM), 80809 Munich, Germany; sebastian.freilinger@tum.de (S.F.); lisa.baumgartner@tum.de (L.B.); tobias.engl@tum.de (T.E.); maximilian.dettenhofer@tum.de (M.D.); steffi.huber@tum.de (S.H.); frauke.muehlbauer@tum.de (F.M.); renate.oberhoffer@tum.de (R.O.)

**Keywords:** pediatric exercise science, young athletes, antioxidative capacity, endothelial health, superoxide dismutase, glutathione peroxidase, physical activity, training adaptation, oxidative stress

## Abstract

Oxidative stress is a key driver of endothelial dysfunction and early cardiovascular risk. Antioxidant enzymes such as superoxide dismutase (SOD) and glutathione peroxidase (GPX) are vital for vascular protection, especially during growth. While exercise-induced redox adaptations are well established in adults, data in pediatric athletes are limited. This cross-sectional study examined associations between training load and systemic antioxidant enzyme activity in 203 youth athletes aged 10–16 years, also considering sex, age, sports discipline, and redox phenotypes. Physical activity was assessed via validated questionnaires and expressed as weekly hours and MET-hours. Fasting blood samples were analyzed for SOD and GPX. Statistical tests included *t*-test, ANOVA, regression, and k-means clustering. Antioxidant enzyme levels were stable across training volumes, sports disciplines, and age groups. Boys showed significantly higher SOD than girls (259.43 ± 54.02 U/mL vs. 226.93 ± 48.22 U/mL, *p* < 0.001); GPX levels were similar between sexes. Cluster analysis identified three distinct redox profiles with differing training and sex distributions. No linear association was observed between training load and enzyme activity. Findings suggest that youth athletes exhibit robust antioxidant defenses, with individual and sex-related factors playing a more prominent role than training volume. These results highlight the value of regular physical activity for vascular health during development and the need for longitudinal studies to track redox adaptation.

## 1. Introduction

Oxidative stress is broadly defined as an imbalance between the production of reactive oxygen species (ROS) and the capacity of antioxidant defense [1]. When ROS generation overwhelms antioxidant systems, the excess ROS damage cellular lipids, proteins, and DNA [2]. In the endothelium, elevated ROS trigger lipid peroxidation, inflammation and rapid NO inactivation, reducing NO bioavailability and impairing endothelium-dependent vasodilation, resulting in endothelial dysfunction, an early hallmark of vascular pathology and atherogenesis [3,4,5]. Notably, such dysfunction appears even in children and adolescents, especially those with cardiovascular risk factors, indicating that the roots of atherosclerotic disease can be present at a very young age [6]. Hence, effective antioxidant defenses in youth are critical to preserve endothelial function and vascular health and should further be strengthened through appropriate measures, such as regular physical activity or athletic training [7].

Antioxidant enzymes, particularly superoxide dismutase (SOD) and glutathione peroxidase (GPX), are key defenses against endothelial ROS [8]. SOD converts superoxide (O_2_^−^) into O_2_ and H_2_O_2_, preventing direct oxidative damage and formation of peroxynitrite (OHOO^−^) from NO. GPX then reduces H_2_O_2_ to water using reduced glutathione (GSH), producing glutathione disulfide (GSSG) and limiting downstream oxidative stress [9]. Together they preserve redox balance, maintain NO bioavailability, and support normal endothelium-dependent vasomotor function [10,11].

At the molecular level, distinct isoforms of SOD and GPX provide compartmentalized antioxidant protection. SOD1 (Cu/Zn-SOD) is predominantly cytosolic, SOD2 (Mn-SOD) resides in the mitochondrial matrix, and SOD3 (Ec-SOD) is secreted and anchored to the extracellular matrix of the endothelium [12]. In humans, the GPX family comprises eight isoforms (GPX1–GPX8). GPX1 is the abundant, ubiquitously expressed form found primarily in the cytosol, GPX2 is enriched in the intestinal epithelium to protect the mucosal barrier and limit inflammation, and GPX3 is predominantly present in plasma and mainly produced by renal tubular epithelial cells [13]. GPX4 is unique in directly reducing membrane lipid hydroperoxides, preventing lipid peroxidation and ferroptosis, an iron-dependent cell death [14,15]. These isoforms thereby form a layered network that intercepts ROS in different cellular compartments and chemical forms, safeguarding endothelial integrity and NO-signaling.

The expression and activity of SOD and GPX are tightly regulated by redox-sensitive signaling cascades. Upstream kinases such as MAPK, AMPK, or CaMK modulate transcriptional programs in response to fluctuations in ROS, shear stress, and metabolic changes [16,17,18,19]. They feed into transcriptional regulators, mainly Nrf2, NF-κB, and KLF2. Under oxidative stress, Nrf2 dissociates from its inhibitor Keap1 and translocates to the nucleus, where it drives an ARE-dependent antioxidant program [20]. NF-κB, though classically pro-inflammatory, can in certain contexts induce adaptive antioxidants such as the mitochondrial SOD2, tempering collateral oxidative injury during inflammation [21]. Laminar shear stress elevates KLF2, which promotes eNOS and antioxidant gene expression, suppresses NF-κB, and enhances Nrf2 activity [22]. These factors coordinate the dynamic antioxidant response of endothelial cells to redox cues, tuning adaptive defense while maintaining normal signaling.

Acute and chronic exercise elicit complementary effects on endothelial redox defense. A single moderate bout increases shear stress and ROS, rapidly activating Nrf2 and KLF2 [20,23], which triggers post-translational activation of SOD1 [24], early SOD3 transcription [25], delayed induction of SOD2 (via FoxO3a/Nrf2) [26,27], and transient GPX1 and GPX4 fluctuations as GSH is consumed and replenished [28]. This yields immediate superoxide clearance followed by peroxide detoxification, with net benefit depending on exercise intensity [29]. With repeated training, these signals stabilize a more robust redox phenotype, characterized by sustained shear/ROS that keep KLF2 and Nrf2 active, enhanced eNOS/NO output, and improved mitochondrial quality via AMPK/PGC-1α/SIRT3 [30]. This results in persistently elevated SOD1, enhanced SOD2 function, strong SOD3 upregulation, increased GPX1 activity and GPX4 expression with indirect support by greater GSH availability [31,32,33,34,35].

The antioxidant response to exercise is well characterized in adults but remains understudied and inconsistent in children and adolescents, likely due to ongoing maturation of redox systems and heterogeneity in study designs (sports discipline, training volume, developmental stage, sampling time) [36]. Some pediatric cohorts show higher total antioxidant capacity and elevated GPX in active versus sedentary or obese peers, and youth athletes often exhibit more favorable systemic redox profiles [37,38], yet enzyme specific patterns vary. Certain studies have reported increased GPX activity accompanied by reduced SOD levels in trained youth [39], while others have found the inverse pattern, particularly in endurance-trained or team-sports athletes, where SOD activity appeared to be more responsive to training stimuli than GPX [40]. Taken together, these findings suggest exercise influences antioxidant status in youth, but the direction, magnitude and determinants of SOD and GPX activity remain unclear, especially the dose–response with habitual training load and potential modification by age, sex and sports discipline.

To address this gap, this study aims to examine how weekly training load relates to systemic antioxidant enzyme activity by SOD and GPX in 203 competitive young athletes aged 10–16 years. We also explored moderating effects of age, sex and sports discipline to identify subgroup-specific patterns and developmental trajectories.

## 2. Results

### 2.1. Participant Characteristics

The study included 203 young athletes (mean age 13.49 ± 1.64 years) with boys being about half a year older than girls on average. Participants represent 20 sports, the most common being ice hockey (*n* = 52), soccer (*n* = 49), and swimming (*n* = 44). The remaining disciplines include basketball (*n* = 11), field hockey (*n* = 7), track and field (*n* = 7), rowing (*n* = 6), synchronized swimming (*n* = 6), wrestling (*n* = 4), handball (*n* = 4), tennis (*n* = 3), rugby (*n* = 2), wakeboard (*n* = 1), cycling (*n* = 1), triathlon (*n* = 1), biathlon (*n* = 1), golf (*n* = 1), kickboxing (*n* = 1), mountain biking (*n* = 1), and volleyball (*n* = 1). None of the participants reported smoking.

Overall, mean anthropometry was 165.58 ± 12.72 cm in height and 52.51 ± 12.33 kg in weight, corresponding to a BMI of 18.85 ± 2.27 kg/m^2^ and a body surface area of 1.54 ± 0.24 m^2^. On average, athletes trained 9.78 ± 4.14 h per week, accumulating 96.89 ± 46.07 MET-h/week. Blood analyses yielded mean SOD and GPX concentrations of 251.91 ± 54.38 U/mL and 278.11 ± 82.16 U/L, respectively. An overview of the study characteristics can be seen in Table 1.

Comparing sexes, boys and girls had similar training loads (hours and MET-hours), but boys were significantly taller (*p* = 0.005) and had a larger BSA (*p* = 0.022) than girls. Boys also exhibited higher antioxidant activity as measured by SOD (*p* < 0.001), whereas GPX levels did not differ significantly between sexes.

### 2.2. Age and Maturation Group Comparisons

To assess developmental effects, athletes were divided at the median age (13.84 years) into younger (*n* = 101) and older (*n* = 102) groups (Table 2). Older athletes were significantly taller and heavier than younger ones (both *p* < 0.001) and therefore had higher BMI and BSA (*p* < 0.001 for both). Older athletes also reported greater weekly training hours and higher training intensity (both *p* < 0.001). In contrast, mean SOD levels were comparable across age groups, and although younger athletes tended to have lower GPX, this difference was not statistically significant. To complement the age-based classification, the cohort was additionally stratified at the median BSA value into lower-BSA and higher-BSA subgroups (Table 3). Athletes above the BSA median were significantly taller and heavier and exhibited higher BMI and BSA values (all *p* < 0.001), confirming the expected morphological differences. Interestingly, while athletes with higher BSA continued to show slightly elevated training hours and MET-based intensity, these differences were no longer statistically significant. Antioxidative enzymes again did not show significant differences between BSA-based groups.

### 2.3. Training Context: Club-Only vs. Additional Extracurricular Sport

Athletes were classified into those engaged only in organized club training (*n* = 110) versus those with additional extracurricular exercise (*n* = 83) (Table 4). The groups were similar in all respects except training duration: extracurricular athletes spent significantly more time training per week than club-only athletes (*p* < 0.001). SOD concentrations did not differ between groups; GPX was slightly higher in the club-only group, but this difference did not reach significance.

#### Sport-Specific Differences Among Male Athletes (Soccer, Ice-Hockey, Swimming)

Only male athletes were included in these sport-specific analyses because the number of female participants in soccer and ice hockey was too low to allow meaningful comparisons across genders. A one-way ANOVA in the three largest sport-specific subgroups (soccer, swimming, ice hockey) showed no significant differences in age or anthropometrics across sports (Table 5). We used an unadjusted ANOVA to estimate the total between-sport effect, while acknowledging potential residual confounding. Accordingly, we also performed an adjusted regression as a robustness check. Swimmers trained substantially more than the other groups, reporting the highest weekly hours and MET-hours (*p* < 0.001 versus the other disciplines). Neither SOD nor GPX differed significantly among the sports, although swimmers tended to have the lowest SOD levels and ice-hockey players the highest GPX levels.

Across the disciplines, the subjects did not differ significantly in terms of age and anthropometric parameters. Swimmers had the highest amount of weekly training load concerning both training duration and intensity, significantly differing from the other two disciplines. SOD and GPX values remained statistically insignificant across all three groups, wherein swimmers showed the lowest SOD levels and ice-hockey players the highest GPX concentrations.

### 2.4. Association of Training Volume/Intensity with Antioxidative Markers

Multiple linear regression models (total sample and sex-specific), adjusted for age, sex, and BSA, tested whether training duration or intensity predicted antioxidative markers (Table 6 and Table 7). No significant predictors of SOD or GPX were identified. In general, higher training intensity was associated with lower SOD and GPX (non-significant trend), and longer training duration was weakly associated with higher SOD but lower GPX. In boys, both training hours and intensity showed inverse, yet non-significant, relationships with SOD and GPX. In girls, weekly MET-hours showed a negative association with GPX, whereas SOD was positively, though not significantly associated with both MET-hours and training duration.

### 2.5. Cluster-Based Profiles of Training Load and Antioxidant Enzyme Activity

**Figure 1:**
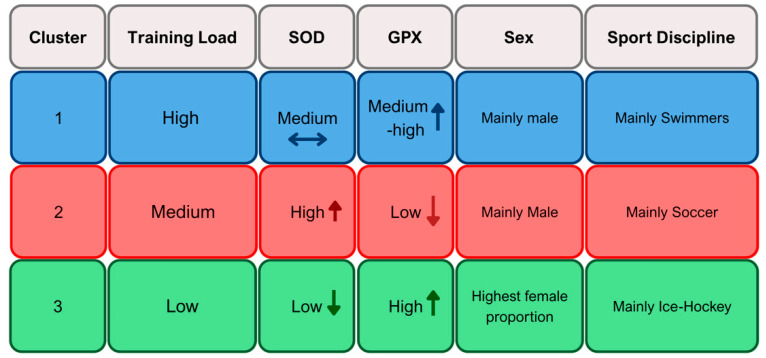
Overview of cluster characteristics.

**Figure 2:**
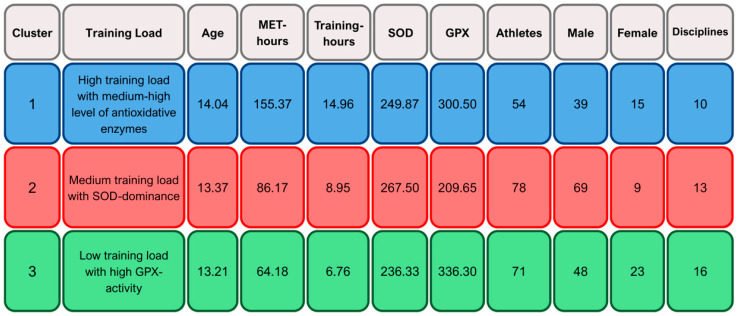
Mean and absolute values of cluster characteristics.

**Figure 3:**
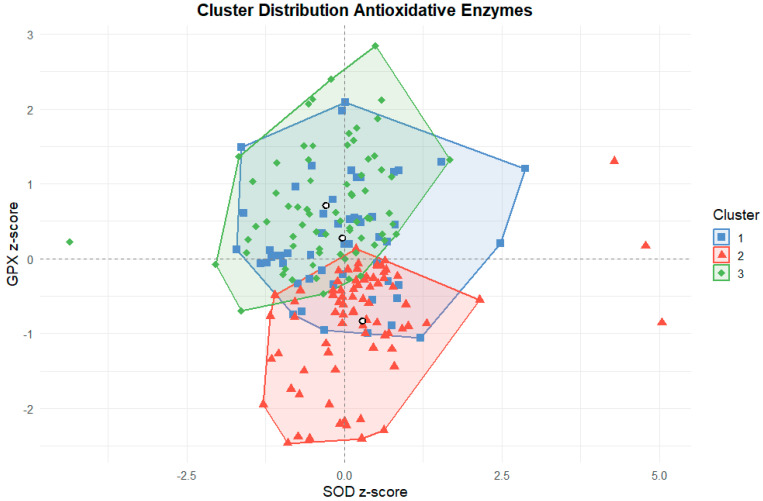
Cluster Distribution across z-values for SOD and GPX.

To identify potential antioxidant adaptation patterns, a k-means cluster analysis was performed using standardized z-scores of SOD, GPX, training hours, and MET-hours. Three distinct clusters emerged (Figure 1 and Figure 2). A graphical distribution across z-scores is displayed in Figure 3.

Cluster 1 (*n* = 54) consisted of athletes with the highest training load (14.96 h/week; 155.37 MET-h/week) and moderate antioxidant enzyme levels (SOD: 249.87 U/mL; GPX: 300.50 U/L). The cluster was predominantly male (*n* = 39) and included a high proportion of swimmers (*n* = 25). The average age was 14.04 years.

Cluster 2 (*n* = 78) was characterized by moderate training load (8.95 h/week; 86.17 MET-h/week), but the highest SOD activity (267.50 U/mL) and the lowest GPX (209.65 U/L). It was largely male (*n* = 69), with many athletes involved in soccer (*n* = 24) and ice hockey (*n* = 19). Mean age was 13.37 years.

Cluster 3 (*n* = 71) had the lowest training volume (6.76 h/week; 64.18 MET-h/week), the highest GPX activity (336.30 U/L), and the lowest SOD (236.33 U/mL). This group had a comparatively higher proportion of female athletes (*n* = 23) and included a broader distribution of sports disciplines (*n* = 16). Mean age was 13.21 years.

## 3. Discussion

This study examined how physical activity influences key antioxidant enzymes in a special cohort of young competitive athletes. Overall, we found that intrinsic factors like sex had a more pronounced association with antioxidant enzyme levels than extrinsic factors such as training load or sports discipline. Notably, the values largely fell within the laboratory specific reference ranges (SOD: 164–240 U/mL; GPX: 200–625 U/L).

### 3.1. Sex Differences

Boys exhibited significantly higher SOD activity than girls, whereas GPX activity was comparable. This is notable, as previous studies typically report minimal sex differences in oxidative markers among children and adolescents, and adult females often show higher antioxidant capacities [41]. This may be attributed to the protective effects of estrogen and other female hormones on the redox system [42,43]. The observed elevated SOD in boys may reflect advanced pubertal status within the male subgroup. Hormonal modulation may also be involved, as testosterone has been shown to stimulate SOD production [44], whereas estrogen typically upregulates GPX [45]. The moderate enzyme levels observed across both sexes likely result from age-related hormonal influences being partly offset by regular physical activity, maintaining a balanced antioxidant profile at this developmental stage.

### 3.2. Maturation and Age

Age had no significant effect on SOD and GPX levels, despite higher training intensity in older athletes (>13.84 years). Although adolescence typically enhances antioxidant capacity [46], our findings suggest that regular training may already induce maximal adaptation, minimizing age-related differences [40,47]. The high adaptability of the pediatric antioxidant system may mask developmental effects, as even younger athletes likely surpass sedentary peers. Additional analysis by BSA showed size differences without corresponding changes in enzyme levels [48], indicating that biological maturation, not body size alone, influences antioxidant capacity.

### 3.3. Training Load

No significant linear relationship was found between training duration or intensity and SOD or GPX. Neither weekly training hours nor MET-hours predicted enzyme activity. This may reflect a plateau effect, where athletes have already surpassed the threshold for antioxidant adaptation, leading to stable resting levels. However, differences might emerge during acute exercise or recovery, as trained individuals often show faster antioxidant responses [49]. The observed stability likely reflects homeostatic regulation within a narrow range, potentially critical for maintaining endothelial NO bioavailability under stress. While resting levels were similar, functional antioxidant capacity may still vary, highlighting the need for longitudinal and dynamic assessment. It should be noted that our study did not include a non-exercising control group for direct comparison. Prior research indicates that youth athletes generally display higher antioxidant capacity than age-and sex-matched sedentary peers, even when dietary antioxidant intake and plasma vitamin levels do not differ, supporting the notion that the consistently stable enzyme activity observed in our cohort likely reflects an already elevated baseline status [50].

### 3.4. Sport Discipline

No Sport-specific differences in SOD and GPX were found among soccer, swimming and ice hockey athletes, despite swimmers having higher training volumes. While endurance sports typically enhance oxidative metabolism [51], all three disciplines involve mixed aerobic demands, possibly leading to similar antioxidant adaptations. This suggests that structured physical activity, regardless of sport, promotes a comparable antioxidant defense at rest. From a health perspective, this is encouraging, as it indicates no elevated oxidative risk in any one sport. Common factors such as nutrition, recovery, practices, and general fitness may also contribute to these uniform profiles. However, sample size and sex distribution limitations may have masked smaller sport specific effects.

### 3.5. Antioxidant Clusters and Redox Phenotypes

Cluster analysis identified three distinct athlete profiles based on training load, antioxidant enzyme activity, sex and sport. Cluster 1 had the highest training volume, moderate-to-high enzyme levels, mainly male swimmers, and the oldest average age. Cluster 2 showed moderate training, high SOD and low GPX, and included mostly male soccer and ice hockey players. Cluster 3 had the lowest training load, high GPX and low SOD and the highest female representation and sport diversity.

These patterns likely reflect sex-related differences, as females typically show stronger glutathione-based defenses. The elevated GPX in Cluster 1, despite its male dominance, may indicate a compensatory response to high oxidative stress from endurance training, particularly in swimmers. Thus, extreme training loads might outweigh typical sex-based antioxidant profiles.

The identification of these clusters could indicate the existence of distinct redox phenotypes among young athletes. Physiologically, this implies variability in individual antioxidant responses to similar training stimuli, suggesting some athletes develop strong antioxidant defenses, whereas others maintain comparatively lower enzyme levels despite equivalent training exposure. This variability highlights the need for personalized antioxidant profiling, as previously recommended by Margaritelis et al. [52]. Our findings reinforce this concept: identifying subgroups based on antioxidants and training profiles could enable more tailored intervention strategies. Athletes identified within a lower antioxidant cluster might benefit from targeted nutritional interventions (e.g., antioxidant-rich diets) or modified training approaches aimed at reducing oxidative stress risk. Conversely, those with naturally higher antioxidant capacities may require alternative support strategies. Genetic and dietary factors, which were uncontrolled in our analysis, can substantially influence antioxidant enzyme levels. Genetic polymorphisms in antioxidant enzyme genes are known to affect enzyme activity, for example, certain SOD2 variants can alter the efficiency of SOD against superoxide stress [53]. Similarly nutrient status can shape overall antioxidant status, as vitamin intake and other dietary components can modulate antioxidative capacity [50]. Thus, the use of clustering provides a practical, data-driven approach toward individualizing athlete training programs and nutritional guidance.

In summary, baseline antioxidant enzyme levels in youth athletes appear more influenced by sex and individual factors than by age or sport. Females showed higher GPX, males higher SOD, likely due to hormonal regulation. No clear link between training load and enzyme levels was found, suggesting saturation and strong inter-individual variability. The absence of sport-specific differences implies similar oxidative adaptations across disciplines. Clustered redox profiles highlight distinct phenotypes with relevance for personalized training and nutrition. Throughout, the importance of SOD and GPX in vascular health cannot be overstated: by scavenging superoxide and hydrogen peroxide, these enzymes protect endothelial function and maintain vascular tone [11]. Ensuring that adolescent athletes have adequate antioxidant defenses—through appropriate training, diet, and recovery—is therefore critical to prevent endothelial dysfunction and promote lifelong cardiovascular health.

### 3.6. Limitations

The cross-sectional design limits causal inference and prevents conclusions about training-induced changes over time. Antioxidant enzymes were measured at a single time point without a non-athletic control group, restricting our ability to contextualize the athletes’ enzyme levels relative to inactive peers, making our conclusions reliant on comparisons with existing literature, which provides few reliable normative values for this young age group. Additionally, our assessment of physical activity relied on reported training hours and estimated intensity. While we attempted to quantify training load, these measures may not capture all aspects of exercise dose (e.g., intensity distribution, muscle damage, recovery status) and are subject to reporting bias. Factors such as training modality, recent acute exercise bouts, or recovery status may have influenced antioxidant levels but were not accounted for.

Moreover, only resting SOD and GPX levels were measured, offering a static view that may overlook dynamic responses to exercise. In addition, the lack of significant enzyme variation across sports may also reflect the limited sensitivity of SOD and GPX to detect subtle, sport-specific adaptations. Future studies with larger cohorts and broader biomarker panels are needed to address this aspect conclusively. Biological maturation was not directly assessed, although age and body surface area were used as proxies. Individual variability in pubertal stage could have affected enzyme activity. A more accurate measure, such as Tanner staging or hormonal profiling would have strengthened our analysis. We therefore emphasize the need for future studies to include direct hormonal profiling. Finally, genetic and dietary factors were not controlled for. Polymorphisms in SOD or GPX genes and variations in the intake of antioxidant-rich foods or essential micronutrients (e.g., selenium, zinc, copper) may have contributed to inter-individual differences. Including additional markers such as hemoglobin, ferritin, hs-CRP, glutathione and ICAM-1 in future research would also provide a more comprehensive understanding of oxidative stress, inflammation and endothelial health. It should also be noted that the k-means clustering applied was exploratory and lacks external validation, which may increase the risk of overfitting. Future validation in independent cohorts is required to confirm these described phenotypes. Despite these limitations, the study provides a valuable snapshot of antioxidant status in the special cohort of youth athletes and highlights several considerations for future research.

## 4. Materials and Methods

The present cross-sectional analysis is based on baseline data from the first year of the MuCAYAplus study (Munich Cardiovascular Adaptations in Young Athletes Plus) [54], a prospective single-center cohort study conducted at the Institute of Preventive Pediatrics, Technical University of Munich (TUM). The overarching goal of the three-year longitudinal study is to investigate the cardiovascular and systemic adaptations to physical activity in youth athletes. All examinations, including clinical assessments and laboratory analyses, were performed at the study center. The data analyzed in this paper were collected between November 2023 and November 2024. A detailed study protocol describing all methods and procedures has already been published [54].

### 4.1. Participants

Participants were children and adolescents (10–16 years) presenting for sports medical examinations at the outpatient clinic of the Institute of Preventive Pediatrics. Participants were required to meet several inclusion criteria to be enrolled in the MuCAYAplus study. Eligible individuals engaged regularly in a primary sports discipline, were club-affiliated, competed athletically, and trained at least three hours per week. Assessments were conducted at least 12 h after the last intensive session. Inclusion also required annual participation over three years. Exclusion criteria included acute infections, injuries, chronic diseases, or missing consent.

### 4.2. Ethical Considerations

The study protocol was approved by the Ethics Committee of the Technical University of Munich (Approval Number: 516547656). All participants and their legal guardians received both written and verbal information about the study prior to enrollment. Written informed consent was obtained from participants and their legal guardians in accordance with age-appropriate ethical standards.

### 4.3. Venous Blood Sampling and Laboratory Analyses

Fasting venous blood samples were obtained from each participant in the morning hours under standardized conditions. The collection was conducted by trained physicians. The samples were processed and analyzed by an external accredited laboratory (SYNLAB MVZ Labor, Munich, Germany). Quantification of enzymatic antioxidant markers included the activity levels of SOD and GPX, measured photometrically in plasma using standardized, quality-controlled procedures. SOD was measured based on the in vitro generation of superoxide radicals by the xanthine/xanthine oxidase system. These radicals react with the tetrazolium salt I.N.T. (p-iodotetrazolium) to form a red formazan dye. The presence of SOD inhibits this reaction by catalyzing the dismutation of the highly reactive superoxide radicals, thereby reducing dye formation. GPX activity was determined from hemolysates using a coupled enzymatic assay. GPX catalyzes the oxidation of GSH by cumene hydroperoxide to form oxidized GSSG. In the presence of glutathione reductase and NADPH, GSSG is immediately reduced back to GSH, while NADPH is oxidized to NADP^+^. GPX in its oxidized form is rapidly inactivated by cyanide-containing Drabkin’s reagent, the samples were first diluted with a stabilizing buffer to maintain GPX in its reduced form. Drabkin’s reagent was then added to inactivate other peroxidases, which would otherwise interfere with the assay and yield falsely elevated GPX activity values.

### 4.4. Physical Activity Assessment (MoMo-PAQ)

Physical activity (PA) and motor performance levels of participants were evaluated using the validated “Motorik-Modul” Physical Activity Questionnaire (MoMo-PAQ), as established by Bös et al. [55] and Jekauc et al. [56]. This questionnaire provided detailed insights into the participants’ training history, primary sports discipline, weekly training duration, and competition participation. To standardize activity levels across sports, metabolic equivalent task hours (MET-h/week) were calculated, allowing for comparable quantification of activity intensity and duration.

### 4.5. Statistics

Statistical analyses were conducted using SPSS v29.0.2.0 (IBM Corp., Chicago, IL, USA), with significance set at *p* < 0.05. Continuous variables are reported as mean ± SD, categorical data as frequencies (n, %). Group differences by sex and age were tested using *t*-tests, differences across sports via One-Way ANOVA. Associations between training variables and antioxidative enzymes were analyzed using multiple linear regression, adjusted for age, sex, and BSA. We applied exploratory k-means clustering to uncover latent training-redox-phenotypes using standardized z-scores of selected variables. Clusters were then externally profiled by sex, age and sports discipline to derive interpretable phenotypes.

## 5. Conclusions

This study shows that regular physical activity in youth aged 10–16 is associated with stable systemic antioxidant enzyme levels of SOD and GPX, regardless of training volume, sports discipline, age or body size. A significant difference emerged only for SOD, which was higher in boys, suggesting hormonal and maturational influences on redox regulation. Given the role of SOD and GPX in preserving endothelial function by protecting nitric oxide from oxidative inactivation, their stability across training contexts may contribute to early vascular health and protection against endothelial dysfunction, a risk factor for cardiovascular disease. The observed sex-specific difference in SOD further highlights the complex interaction of hormonal and maturational factors in redox regulation during puberty.

Our cluster analysis revealed individual variability in antioxidant profiles despite similar training exposure, supporting the notion of distinct redox phenotypes. This suggests the potential value of personalized training and nutrition strategies to maintain optimal redox and endothelial function. While the cross-sectional design limits causal conclusions, longitudinal studies are needed to track redox adaptation during adolescence. Such data are essential to guide targeted interventions and youth-specific training guidelines. In conclusion, encouraging regular, age-appropriate physical activity is key to fostering antioxidant resilience and vascular protection in youth. Future longitudinal research will help shape individualized and developmentally appropriate athletic support.

## Figures and Tables

**Table 1:** Study characteristics and antioxidative markers whole cohort.

	N	Total (M ± SD)	N	Boys (M ± SD)	N	Girls (M ± SD)	*p*
Age (yrs)	203	13.49 ± 1.64	156	13.61 ± 1.63	47	13.09 ± 1.61	0.058
Body mass (kg)	203	52.51 ± 12.33	156	53.44 ± 12.58	47	49.44 ± 11.03	0.051
Body height (cm)	203	165.58 ± 12.72	156	166.76 ± 13.29	47	161.66 ± 9.74	**0.005**
BMI (kg/m^2^)	203	18.85 ± 2.27	156	18.90 ± 2.14	47	18.68 ± 2.69	0.556
BSA (m^2^)	203	1.54 ± 0.24	156	1.56 ± 0.24	47	1.48 ± 0.20	**0.022**
Training duration (h/week)	203	9.78 ± 4.14	156	9.78 ± 3.71	47	9.79 ± 5.38	0.996
Training intensity (MET-h/week)	203	96.89 ± 46.07	156	96.28 ± 40.51	47	98.90 ± 61.52	0.785
SOD (U/mL)	203	251.91 ± 54.38	156	259.43 ± 54.02	47	226.93 ± 48.22	**<0.001**
GPX (U/L)	203	278.11 ± 82.16	156	276.11 ± 85.93	47	284.76 ± 68.58	0.528

BMI, body mass index; BSA, body surface area; MET, metabolic equivalent of task; SOD, superoxide dismutase; GPX, glutathione peroxidase. Significant results are marked in bold.

**Table 2:** Study characteristics and antioxidative markers in younger vs. older athletes.

	N	Under Median (*n* = 101; 72 Boys, 29 Girls) (M ± SD)	N	Over Median (*n* = 102; 84 Boys; 18 Girls) (M ± SD)	*p*
Age (yrs)	101	12.14 ± 1.17	102	14.83 ± 0.64	**<0.001**
Body mass (kg)	101	45.00 ± 9.95	102	59.95 ± 9.69	**<0.001**
Body height (cm)	101	157.38 ± 10.40	102	173.70 ± 9.09	**<0.001**
BMI (kg/m^2^)	101	17.95 ± 2.19	102	19.75 ± 1.99	**<0.001**
BSA (m^2^)	101	1.39 ± 0.19	102	1.69 ± 0.17	**<0.001**
Training duration (h/week)	101	8.78 ± 3.87	102	10.77 ± 4.18	**<0.001**
Training intensity (MET-h/week)	101	84.71 ± 41.41	102	108.95 ± 47.45	**<0.001**
SOD (U/mL)	101	252.32 ± 55.33	102	251.50 ± 53.69	0.914
GPX (U/L)	101	269.86 ± 85.79	102	286.29 ± 77.97	0.155

BMI, body mass index; BSA, body surface area; MET, metabolic equivalent of task; SOD, superoxide dismutase; GPX, glutathione peroxidase. Significant results are marked in bold.

**Table 3:** Study characteristics and antioxidative markers stratified by BSA.

	N	Under Median (*n* = 101; 71 Boys, 30 Girls) (M ± SD)	N	Over Median (*n* = 102; 85 Boys; 17 Girls) (M ± SD)	*p*
Age (yrs)	101	12.43 ± 1.49	102	14.54 ± 0.98	**<0.001**
Body mass (kg)	101	42.24 ± 6.62	102	62.65 ± 7.23	**<0.001**
Body height (cm)	101	155.52 ± 8.23	102	175.54 ± 7.42	**<0.001**
BMI (kg/m^2^)	101	17.39 ± 1.79	102	20.30 ± 1.71	**<0.001**
BSA (m^2^)	101	1.34 ± 0.13	102	1.74 ± 0.12	**<0.001**
Training duration (h/week)	101	9.24 ± 4.18	102	10.32 ± 4.05	0.064
Training intensity (MET-h/week)	101	91.02 ± 47.13	102	102.70 ± 44.47	0.071
SOD (U/mL)	101	248.62 ± 57.11	102	255.17 ± 51.61	0.393
GPX (U/L)	101	268.18 ± 80.60	102	287.95 ± 82.90	0.087

BMI, body mass index; BSA, body surface area; MET, metabolic equivalent of task; SOD, superoxide dismutase; GPX, glutathione peroxidase. Significant results are marked in bold.

**Table 4:** Study characteristics and antioxidative markers club-only vs. additional sport.

	N	Only Club (*n* = 120; 89 Boys, 31 Girls) (M ± SD)	N	Club + Extra (*n* = 83; 67 Boys, 16 Girls) (M ± SD)	*p*
Age (yrs)	120	13.52 ± 1.56	83	13.46 ± 1.75	0.818
Body mass (kg)	120	51.78 ± 12.08	83	53.57 ± 12.68	0.309
Body height (cm)	120		83	166.22 ± 13.45	0.556
BMI (kg/m^2^)	120	18.71 ± 2.36	83	19.06 ± 2.15	0.288
BSA (m^2^)	120	1.53 ± 0.23	83	1.56 ± 0.24	0.358
Training duration (h/week)	120	9.09 ± 3.97	83	10.79 ± 4.19	**0.004**
Training intensity (MET-h/week)	120	92.84 ± 47.02	83	102.73 ± 44.30	0.133
SOD (U/mL)	120	249.42 ± 57.20	83	255.50 ± 50.15	0.435
GPX (U/L)	120	284.64 ± 82.13	83	268.68 ± 81.78	0.174

BMI, body mass index; BSA, body surface area; MET, metabolic equivalent of task; SOD, superoxide dismutase; GPX, glutathione peroxidase. Significant results are marked in bold.

**Table 5:** Discipline-specific differences among male young athletes.

	N	Soccer (M ± SD)	N	Ice-Hockey (M ± SD)	N	Swimming (M ± SD)	*p*
Age (yrs)	46	13.40 ± 1.95	50	13.89 ± 1.30	27	13.07 ± 1.63	0.098
Body mass (kg)	46	49.71 ± 14.01	50	55.41 ± 10.78	27	50.42 ± 11.54	0.107
Body height (cm)	46	164.21 ± 15.23	50	168.36 ± 11.40	27	165.80 ± 12.88	0.210
BMI (kg/m^2^)	46	18.38 ± 2.33	50	19.35 ± 1.96	27	18.56 ± 2.09	0.073
BSA (m^2^)	46	1.51 ± 0.27	50	1.60 ± 0.20	27	1.50 ± 0.22	0.108
Training duration (h/week)	46	9.77 ± 3.24	50	8.48 ± 3.18	27	12.90 ± 4.41	**<0.001**
Training intensity (MET-h/week)	46	97.18 ± 32.53	50	80.25 ± 28.93	27	139.05 ± 52.85	**<0.001**
SOD (U/mL)	46	264.71 ± 61.68	50	268.96 ± 58.69	27	245.77 ± 39.64	0.216
GPX (U/L)	46	271.54 ± 82.75	50	292.06 ± 89.11	27	283.96 ± 78.97	0.473

BMI, body mass index; BSA, body surface area; MET, metabolic equivalent of task; SOD, superoxide dismutase; GPX, glutathione peroxidase. Significant results are marked in bold.

**Table 6:** Association between training intensity (MET-h/week), training duration (hours/week) and antioxidative markers.

	β	Std. β	*p*	R^2^
MET-h/week				
SOD (U/mL)	−0.035	−0.030	0.680	0.070
GPX (U/L)	−0.031	−0.018	0.810	0.028
Hours/week				
SOD (U/mL)	0.045	0.003	0.962	0.070
GPX (U/L)	−0.363	−0.018	0.802	0.028

SOD, superoxide dismutase; GPX, glutathione peroxidase.

**Table 7:** Sex-differences in the association between training intensity (MET-h/week), training duration (hours/week) and antioxidative markers.

	Boys				Girls			
	β	Std. β	*p*	R^2^	β	Std. β	*p*	R^2^
MET-h/week								
SOD (U/mL)	−0.079	−0.059	0.483	0.018	0.047	0.060	0.709	0.017
GPX (U/L)	−0.028	−0.013	0.873	0.030	−0.027	−0.025	0.879	0.014
Hours/week								
SOD (U/mL)	−0.380	−0.026	0.754	0.016	1.029	0.114	0.479	0.025
GPX (U/L)	−0.781	−0.034	0.683	0.031	0.506	0.040	0.807	0.015

SOD, superoxide dismutase; GPX, glutathione peroxidase.

## Data Availability

The datasets presented in this article are not readily available due to privacy and ethical restrictions. Requests to access the datasets should be directed to the corresponding author.

## Abbreviations

The following abbreviations are used in this manuscript:

ROS	reactive oxygen species
DNA	deoxyribonucleic acid
NO	nitric oxide
SOD	superoxide dismutase
GPX	glutathione peroxidase
O_2_^−^	superoxide anion
H_2_O_2_	hydrogen peroxide
OHOO^−^	peroxynitrite
GSH	reduced glutathione
GSSG	glutathione disulfide
MAPK	mitogen-activated protein kinase
AMPK	AMP-activated protein kinase
CaMK	Ca^2+^/Calmodulin-dependent kinase
Nrf2	nuclear factor erythroid 2-related factor 2
NF-κB	nuclear factor kappa B
KLF2	Kruppel-like factor 2
ARE	antioxidant response element
eNOS	endothelial nitric oxide synthase
FoxO3a	forkhead transcription factor 3a
SIRT3	sirtuin 3
PGC-1α	peroxisome proliferator-activated receptor gamma coactivator 1α
MuCAYAplus	Munich Cardiovascular Adaptations in Young Athletes Plus
MoMo-PAQ	Motorik-Modul-Physical Activity Questionnaire
MET	metabolic equivalent of task
BSA	body surface area
BMI	body mass index
